# A methodology for the production of microfabricated electrospun membranes for the creation of new skin regeneration models

**DOI:** 10.1177/2041731418799851

**Published:** 2018-09-21

**Authors:** Ilida Ortega Asencio, Shweta Mittar, Colin Sherborne, Ahtasham Raza, Frederik Claeyssens, Sheila MacNeil

**Affiliations:** 1 Bioengineering and Health Technologies Group, The School of Clinical Dentistry, The University of Sheffield, Sheffield, UK; 2 Biomaterials and Tissue Engineering Group, Department of Materials Science and Engineering, Kroto Research Institute, The University of Sheffield, Sheffield, UK

**Keywords:** Skin, rete ridges, biomimetic, electrospinning

## Abstract

The continual renewal of the epidermis is thought to be related to the presence of populations of epidermal stem cells residing in physically protected microenvironments (rete ridges) directly influenced by the presence of mesenchymal fibroblasts. Current skin in vitro models do acknowledge the influence of stromal fibroblasts in skin reorganisation but the study of the effect of the rete ridge-microenvironment on epidermal renewal still remains a rich topic for exploration. We suggest there is a need for the development of new in vitro models in which to study epithelial stem cell behaviour prior to translating these models into the design of new cell-free biomaterial devices for skin reconstruction. In this study, we aimed to develop new prototype epidermal-like layers containing pseudo-rete ridge structures for studying the effect of topographical cues on epithelial cell behaviour. The models were designed using a range of three-dimensional electrospun microfabricated scaffolds. This was achieved via the utilisation of polyethylene glycol diacrylate to produce a reusable template over which poly(3-hydrroxybutyrate-*co*-3-hydroxyvalerate) was electrospun. Initial investigations studied the behaviour of keratinocytes cultured on models using plain scaffolds (without the presence of intricate topography) versus keratinocytes cultured on scaffolds containing microfeatures.

## Introduction

Skin has a continuously renewing epidermis which acts as a protective surface barrier for the body. While there are several theories of how epidermal stem cells divide and renew to provide skin which lasts a lifetime,^1^ one of the key areas to be explored is the concept of the native skin stem cell niche.^2,3^ Native stem cell niches exist within both embryonic and somatic tissues in vertebrates and invertebrates. These protected and restricted anatomical spaces are thought to be a key feature for understanding how stem cells survive in a relatively quiescent state, physically protected and yet able to give rise to a supply of daughter cells which ensure epidermal renewal throughout a lifetime.^4^

Stem cell niches in the skin are thought to be embedded within the rete ridge areas which play a critical role in maintaining the structure and mechanical properties of the tissue, as well as in directing its regenerative potential. Rete ridges show dimensions ranging from 50 to 400 µm in width and from 50 to 200 µm in depth^1,5,6^ and they are believed to increase the surface area between the dermis and the epidermis, enhancing both the mechanical shear resistance of the skin and the paracrine diffusion between the layers. These micro-topographical structures create distinct cellular microenvironments that differentially direct keratinocyte phenotype and cellular function. Keratinocytes leave the basal layer and differentiate upwards to provide the cornified barrier layers. Some of the specific factors that sustain stemness and regulate keratinocyte differentiation have been thoroughly explored in recent years; it is known that differentiation can be triggered by biophysical elements including shear stress and oxygen tension and it is influenced by paracrine and signalling from stromal fibroblasts.^7^ In order to investigate the role of enclosed three-dimensional (3D) microenvironments on directing skin cell behaviour, several groups have recently developed in vitro models to characterise the effects of cell geometries and surface chemistries on keratinocyte function.^8^ Although these models have provided new evidence, understanding skin cell behaviour within instructive enclosed microenvironments still remains a big challenge.

There is a need for the development of more innovative in vitro models to study skin cell behaviour. The use of engineering methods to produce artificial microfeatures to mimic aspects of the endogenous niche is a useful tool that can provide us with a better understanding of the mechanisms underlying skin renewal. Artificial microenvironments can be produced by different methodologies including template assisted assembly of electrospun fibres,^9–11^ laser-based techniques,^12–14^ electrolysis^15^ or moulding.^16^

Our group has previously reported on methodologies for producing artificial microfeatures for the study of corneal epithelial regeneration^10,17,18^ via a versatile manufacturing method (patented) combining additive manufacturing techniques and electrospinning. In this method, a micropatterned template is fabricated layer-by-layer (in this case with microstereolithography). This template is then used as an electrospinning collector which allows the creation of an electrospun microfabricated mat that reproduces the morphology dictated by the underlying pattern. In this study, we have expanded the use of this patented technology and we have adapted it to the development of 3D microstructured electrospun scaffolds for the study of skin cell interactions. These 3D electrospun scaffolds have been particularly designed so that keratinocyte behaviour can, in the future, be studied in the presence and absence of the stromal fibroblasts (throughout the optimisation of a bilayer design, see Figure 2). Preliminary results present evidence that the presence of the microfeatures positively influences keratinocyte behaviour.

## Materials and methods

### Materials

#### Chemicals and reagents

Tissue culture plastic was purchased from NuncTM (Nalgene, UK). Tissue culture media was purchased from GIBCO (UK). Foetal calf serum was purchased from Biowest Biosera (UK). MTT (3-(4,5-Dimethylthiazol-2-yl)-2,5-diphenyltetrazolium bromide), polyethylene glycol diacrylate (PEGDA) (Mn 250) and camphorquinone were purchased from Sigma (UK). Syto9 and propidium iodide (PI) were purchased from Invitrogen (UK).

Skin was obtained from patients undergoing routine abdominoplasties and breast reductions who gave written informed consent for skin not required for their treatment to be used for research purposes on an anonymous basis. Skin was obtained under a Human Tissue Authority Research Tissue Bank Licence number 12179. This research was also covered by Ethics Committee Approval reference 15/YH/0177. Skin was used to isolate keratinocytes.

### Methods

#### Stereolithography for template production

**Figure 1.**
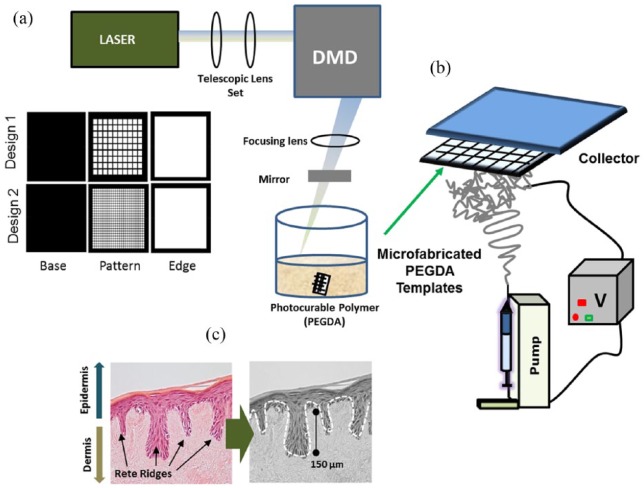
Schematic of the manufacturing of the constructs. Panel (a) shows a schematic of the in-house developed microstereolithography set-up in which a blue laser is focussed into a digital multimirror device via the use of a telescopic lens set; the beam is later directed to a focussing lens followed by a mirror; a bath containing a photocurable polymer (PEGDA) is placed on a xyz stage. Panel (a) also shows a schematic of the individual projected layers for two types of microfeature. Panel (b) shows a schematic of the electrospinning process performed using the PEGDA templates; these templates are attached to a metallic base in order to create electrospinning collectors in which to spin a PHBV solution. Panel (c) shows a histology image of the native Rete Ridges in the skin; this specific image corresponds to a sample of tissue engineered skin produced in our laboratory and exemplifies the type of native topography we aim to emulate in this work.

Stereolithography was used to produce the initial templates using a blue laser beam (blue laser MBL-III 473 nm; 150 mW) focussed into a DMD (digital multimirror device, ultraviolet (UV)-enabled starter kit, Texas, Instruments). Computer-aided designs consisted of three layers including a plain base, a patterned micropocket-like layer and an edge layer (as shown in Figure 1(a)). The reflected 2D laser image of the desired pattern was collected by a 2.5 cm diameter, 10 cm focal length lens (Thorlabs) and reflected downwards by a mirror onto an acetate sheet in a six-well plate containing the photocurable pre-polymer PEGDA (M_n_ 250 g/mol) (Sigma-Aldrich, UK) with 1% camphorquinone used as photoinitiator. The PEGDA and camphorquinone solution were mixed for 30 min prior to use. Using the set-up described, it is possible to manufacture templates of approximately 1.5 cm^2^. A range of micropatterns with varying morphologies and sizes were created for this study. For each case, a base of the template was projected onto an acetate sheet in a multiwell plate containing 700 µL of photocurable polymer mix and irradiated between 15 and 60 s (depending on the chosen design). This created a firm base for the multipocket design to attach to (Figure 1). A defined amount of resin (400 μL) was added at each subsequent step in order to form the microfeatures on the base of the template. Once these templates had formed, the excess PEGDA in the well plate was discarded and washed with 100% isopropanol (IPA). This step was repeated 2–3 times. The templates were left to wash in IPA for 2–3 days in order to completely remove uncured PEGDA and excess photoinitiator. The templates were subsequently washed in PBS, dried and stored dry until use.

#### Electrospinning

**Figure 2.**
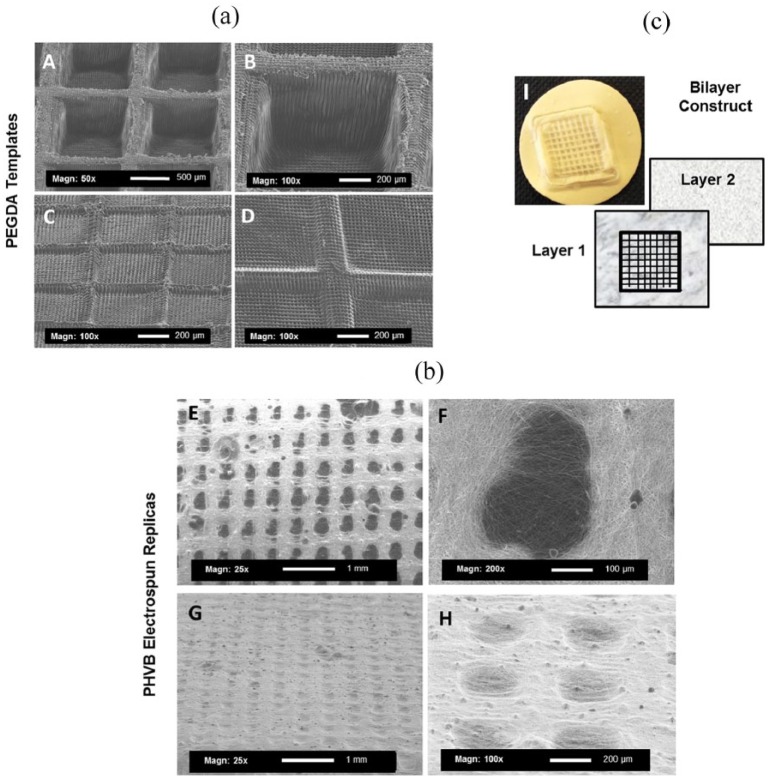
Panel (a) shows examples of optimised PEGDA templates. Images A and B show a rectangular shaped pattern with features with a depth of 500 µm; images C and D show square-shaped morphologies with a depth of 200 µm. Panel (b) shows an example of the electrospun membrane replicas for both square and rectangular patterns (images E–H). Panel (c) shows an image (I) of a microfabricated construct (1.5 cm × 1.5 cm size) containing two layers of electrospun scaffold; layer 1 contains the micropocket pattern and layer 2 is a plain electrospun scaffold covering the lower surface of the microfabricated template (back layer).

The PEGDA templates were fixed using scanning electron microscopy (SEM) carbon tabs on an electrospinning mandrel. Poly(3-hydrroxybutyrate-*co*-3-hydroxyvalerate) (PHBV) (3 g) with 3 g methanol and 24 g dichloromethane (DCM) were dissolved to obtain a 3% (w/v) solution and magnetically stirred overnight to dissolve the bulk polymer. The polymer solutions were separately fed into 4 × 5 mL standard syringes attached to a 21G blunted stainless steel needle using a syringe pump (KDS 100; KD Scientific, Holliston, MA) at a flow rate of 40 µL/min. A high voltage of 17 kV (Gamma High Voltage Research, Ormond Beach, FL) was applied and the polymer solution was spun into fibres and collected on an aluminium foil wrapped collector at a distance of 17 cm from the needle tip to the micropocket templates. The electrospun scaffolds were dried overnight under vacuum. The micropocket electrospun scaffolds were peeled from the aluminium foil and spun on the reverse side. These electrospun mats were then dried under vacuum for 24 h and reversed and electrospun again (using the same solution and the same electrospinning conditions) to provide complete coverage over the back of the micropockets (Figure 2).

#### Isolation and culturing of keratinocytes

Human skin keratinocytes were isolated and harvested from split thickness skin grafts (STSGs) that were obtained from consenting patients undergoing routine breast reduction and abdominoplasties under the Human Tissue Authority (HTA) 12179.

The STSGs were cut into 0.5 cm^2^ pieces using a scalpel blade and incubated overnight with 10 mL of 0.1% w/v Difco trypsin at 4°C. Green’s media consisting of Dulbecco’s Modified Eagle’s Medium (DMEM) and Ham’s F12 medium, supplemented with 10% w/v foetal calf serum (FCS), 100 IU/mL penicillin, 100 µg/mL streptomycin, 0.625 µg/mL amphotericin B, 6.25 µg/mL adenine, 10 ng/mg transferring, 5 µg/mL bovine insulin, 0.4 µg/mL hydrocortisone and 8.5 ng/mL cholera toxin containing FCS were then added to neutralise the trypsin.

Epidermal and dermal layers were carefully separated using forceps and the under-surface of the epidermis and papillary surface of the dermis were gently scraped to remove the keratinocytes. The freshly isolated keratinocytes were collected in Green’s media into a sterile universal tube and centrifuged at 200 g for 5 min. The supernatant was discarded and the keratinocytes were re-suspended in Green’s media for 10–15 times to ensure single cell re-suspension. Keratinocytes were placed into a sterile tissue culture flask (T75) with a feeder layer of lethally irradiated 3T3 (i3T3) cells and cultured at 37°C in 8–10 mL of Green’s media. An irradiated layer of murine3T3 fibroblasts was used to improve the cell culture life-span and allow effective proliferation and differentiation of keratinocytes in vitro.

Isolated keratinocytes were cultured at 37°C in a 5% CO_2_/95% air humidified incubator and re-fed every 2 days with fresh complete media. Keratinocytes were split 1:3 when they reached 80% confluence and sub-cultured. Keratinocytes were used from passage 0. Skin cells were seeded on plain and microfabricated scaffolds at a density of 3 × 105 cells/mL (30,000 per scaffold), then cell performance was analysed using MTT, SEM and live-dead staining (see details below).

#### Assessment of cell viability using an MTT assay

Plain and microfabricated scaffolds (n = 3) were seeded with 30,000 cells per scaffold and studied at 1, 3 and 7 days using MTT assay. For total cellular viability, the media were removed from the wells containing the scaffolds and scaffolds were washed three times with PBS. MTT solution (0.5 mg/mL in PBS) was then added to the scaffolds and placed in an incubator at 37°C for 40 min.

MTT solution was removed and scaffolds imaged. 2-Ethoxyethanol was then added to elute the formazan from the samples. The optical density of the eluted formazan was measured at 540 nm and referenced at 630 nm.

#### Live dead staining

Live dead staining was performed on plain and microfabricated scaffolds using 30,000 cells per scaffold (n = 3). For identification of live and dead cells SYTO9 and PI, solutions were made up as per manufacturer’s guidelines in cell culture medium. The media were removed from the samples and gently washed with PBS. The mixture of SYTO9 (5 µM) and PI (5 µM) in PBS was added to the samples and incubated at 37°C for 15 min. The solution was removed and samples washed with PBS. Samples were visualised using a confocal microscope. The excitation wavelength was 480 nm and emission at 500–550 nm for SYTO9 while for PI the excitation wavelength was 535 nm and emission at 565–617 nm. The sample was visualised using a Zeiss 510 meta confocal upright microscope using Achroplan (water dipping); 10× objective (NA 0.3 WD 2.6 mm) (pin hole adjusted to 1 airy unit, scan speed of 6 with scan average of 4 at 512 × 512 pixel) z-stack images were taken from three independent areas and the number of total cells and dead cells were counted using ImageJ.

#### SEM

##### PEGDA templates

The PEGDA templates were extensively washed in 100% IPA to wash away excess uncured PEGDA. The templates were left in 70% Industrial Methylated Spirit (IMS) for 24 h and stored under dry conditions. The templates were gold coated and imaged using a Philips X-L 20 microscope.

##### Electrospun scaffolds

Plain and microfabricated electrospun scaffolds were fixed at 1, 3 and 7 days and processed for SEM imaging (n = 3). The scaffolds were washed in PBS and fixed in 10% buffered formaldehyde solution for 10–15 min; 0.1 M cacodylate buffer was added and incubated for 20 min. After 20 min, cacodylate buffer was aspirated and 2.5% glutaraldehyde in buffer was added to the samples for 30 min. Post 30 min, glutaraldehyde was aspirated and 1 mL 0.1 M cacodylate buffer was added to rinse off the glutaraldehyde from the surface of the sample, twice for 15 min each. After washing, 1% osmium tetraoxide was added and samples incubated for 2 h. After 2 h, osmium tetraoxide was aspirated and 0.1 M cacodylate buffer was added to the samples and left for 15 min. Cacodylate buffer was aspirated and replaced by 75% ethanol and incubated for 30 min, aspirated and replaced by 95% ethanol for another 30 min. 95% ethanol was aspirated and replaced with 100% ethanol and incubated for another 30 min and subsequently aspirated and replaced with 100% ethanol dried over anhydrous copper sulphate for 30 min. The ethanol was aspirated and hexamethyldisilazane was added to the samples for 30 min and aspirated. The samples were left to dry overnight and sputter-coated with gold for under a vacuum pressure of 0.05 atm, with a current of 15 mA for 2 min in an Emscope SC 500 Coater; the samples were then analysed using a Philips X-L 20 microscope.

#### Statistics

Statistical analyses were performed on GraphPad Prism software using two-tailed Student t-test. In all cases, p values < 0.05 were considered as statistically significant. Please note that the number of scaffolds per each of the reported experiments was 3 (n = 3) and each experiment was repeated three times (N = 3).

## Results

### Fabrication of PEGDA templates

The microstereolithography set-up described above allows the design of ~1.5 cm diameter objects with a minimum resolution of ~50 µm, enabling the construction of a square of micropockets on a base of PEGDA. The manufacture of these microstructured constructs was optimised and square and rectangular shaped PEGDA templates were produced with edge sizes ranging from 200 to 1000 µm and depths varying from 200 to 500 µm (see Figure 2(a)).

PHBV (containing 10% w:w of methanol) was electrospun on the optimised PEGDA templates; SEM imaging showed how the fibres followed the shape of the underlying pattern. A second layer was spun on the back of the microfabricated electrospun scaffold to produce a bilayer structure (see schematic in Figure 2(c)). SEM images and Image J software were used to calculate fibre diameters which were under 1 µm (0.75 ± 0.05 µm) for both layers of the construct.

### Ability of the scaffolds to support cell attachment and cell proliferation

**Figure 3.**
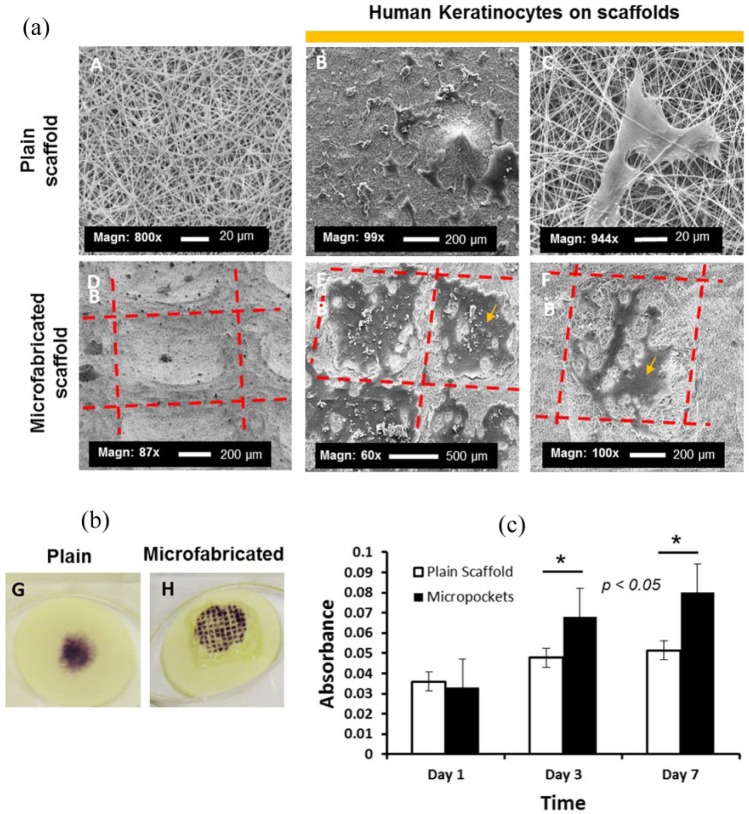
Human keratinocytes growing on microfabricated and plain scaffolds. Panel (a) shows SEM images of keratinocytes attached to both plain and microfabricated scaffolds after 24 h of culture. Panel (b) shows representative MTT assay images highlighting the position of skin cells in both plain and microfabricated scaffolds. Panel (c) shows MTT quantitative data at different time points (1–7 days) comparing plain scaffolds and scaffolds with microfeatures and highlighting significant differences between plain and microfabricated scaffolds for both 3 and 7 days (t student, p < 0.05, N = 3, n = 3).

Keratinocytes were seeded onto electrospun plain and micropocket containing scaffolds. Cells were fixed at different time points and analysed by SEM. Figure 3 shows cells attached to the scaffolds (plain and microfeatured) after 24 h of culture.

Keratinocytes were seeded on both the plain and microfeatured scaffolds and metabolic activity was assessed using MTT. A clear purple colouration of the scaffold was seen, denoting areas in where cells were seeded (Figure 3). Elution of the colour showed that the viability of cells seeded on the patterned scaffolds was significantly greater than on the plain scaffolds (Figure 3(c)).

**Figure 4.**
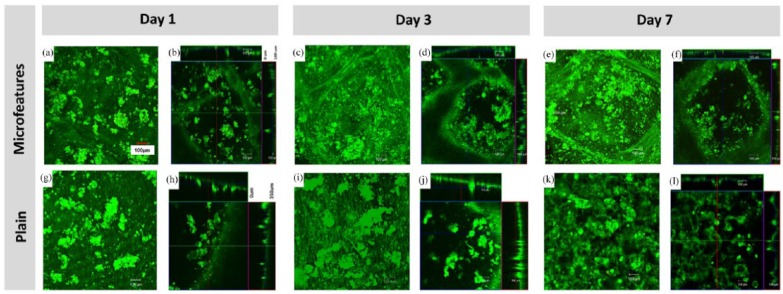
Confocal images and z-stack representations showing live keratinocytes (SYTO9 staining, green) at different time points (1, 3 and 7 days) on both microfabricated (a–f) and plain scaffolds (g–l).

To examine cell viability further, live–dead studies were undertaken using SYTO9 and PI. Keratinocytes were seeded on the scaffolds (30,000 cells per scaffold) and samples were studied at 1, 3 and 7 days of culture. Figure 4 shows confocal and Z-stack images of live cells (SYTO9, green) on both plain and microfabricated scaffolds (the percentage of dead cells was lower than 1% at 7 days). Keratinocytes formed randomly distributed colonies throughout the plain scaffolds whereas there appeared to be more colonies retained within the microfeatures for the microfabricated scaffolds.

## Discussion

Electrospinning has been used extensively by tissue engineers to produce scaffolds for biomedical applications; it is a highly versatile technique in which one can spin fibres of different diameters, different orientations and intermingle fibres so that one can produce bilayer and trilayer, micro- and nano-fabricated scaffolds^19,20^ and even be used to produce electrospun scaffolds with features within them by spinning over a patterned collector.^17^

The desire to produce microfeatures in scaffolds is really stimulated by our increasing understanding of how stem cell niches contribute to the repair and regeneration of damaged tissues throughout our life. Research in this area has focussed on both the study of metabolic and biological cues of the niches environments^21,22^ and the design and manufacture of physical spaces for the control of stem cell fate.^18,23,24^

In our development of patterned scaffolds, these were initially developed to study the impact of microfabricated pockets on the performance of limbal epithelial stem cells for corneal regeneration.^10,17,18^ In these papers, we produced the 3D architecture by microstereolithography and then spun fibres over the resulting template showing that the fibres picked up the gross morphology of the underlying topography. We were able to show that the micropockets enhanced the migration of cells from limbal explants and indeed, these cells transferred readily from the membranes to an ex vivo cornea model.^18^ In some studies we pre-treated the microfabricated structures with biotinylated fibronectin and were able to show that cell outgrowth from fibronectin-coated microfabricated structures was 50% greater than from scaffolds without structures (or from simple fibronectin coating alone).^12^

In this article, we have extended the use of the above technique, applying it to the design of a future cell-free microfabricated and multilayered fibrous membrane for skin regeneration. Specifically, we have demonstrated the ability to design and produce optimised microfeatured electrospun membranes with dimensions in the range of the rete ridges found in the native skin. Including the rete ridge concept within the design of new skin in vitro models is an innovative approach that can provide us with key understanding about skin regeneration mechanisms; for example, skin vulnerability to injury has been related to the lack of structural stability which is ultimately associated with a flattened dermal epithelial junction which generally involves the lack of rete ridge structures.^25^

Our rete ridge-like electrospun membranes have been tested in vitro using human primary keratinocytes and we demonstrate that these cells attach and proliferate on the scaffolds, migrating within the niche-like structures and showing their typical keratinocyte morphology (Figures 3 and 4). Interestingly, when measuring metabolic activity at different time points, it was observed that metabolic activity was higher overtime for cells located on the microfabricated scaffolds than for cells placed on the plain scaffolds. We hypothesise that the increase of surface area provided by the pockets allows cells a bigger area in which to proliferate (Figures 3(b) and (c)); this observation is consistent with previously reported data in which the rete ridges were shown to play a role in increasing the surface area between the dermis and the epidermis, therefore influencing mechanical stability.^2,5,6^

In our current design, we have added a second electrospun layer (back layer) which allows the creation of a more complex model in which we can, in the future, include fibroblasts and study their effect on keratinocyte distribution and fate. Specifically, in our future work, we aim to seed keratinocytes on the microfabricated part of the bilayer and fibroblasts on the back membrane and study cell re-distribution and cell differentiation on the constructs. Our group is currently working on an improved manufacturing route which allows the creation of fine-tuned rete ridge-like structures with very accurate features that can be reproduced via electrospinning; in this sense, future work will also include the study of size effects on the rete ridges performance. Although this preliminary model was designed with a non-biodegradable polymer, we are now working towards the use of polylactide-*co*-glycolide (PLGA), which degrades within weeks/months (depending on the content of glycolic acid). This article sets the basis for the development of more complex models in which to study skin cell behaviour as well as for the design of next generation fibrous cell-free membranes for future clinical use in skin regeneration.

## Conclusion

We describe the development of an innovative bilayered microfabricated electrospun membrane which can be used in the study of skin cell regeneration. This membrane seeks to mimic the epidermal/dermal morphology found in native skin tissue by incorporating well-defined invaginations or micropockets to simulate the rete ridges. Human keratinocytes were cultured in these models and they successfully attached and proliferated on the electrospun membranes. Cells seemed to preferably locate on the niche-like areas and an increase in metabolic activity was observed when keratinocytes were seeded on the microfabricated scaffolds (in comparison with plain (non-structured) counterparts). These membranes are a new tool for studying skin cell interactions and will hopefully provide key data for the creation of cell-free new skin regenerative membranes.
